# Hospital indoor air quality and its relationships with building design, building operation, and occupant-related factors: A mini-review

**DOI:** 10.3389/fpubh.2022.1067764

**Published:** 2022-11-08

**Authors:** Farha Ibrahim, Ely Zarina Samsudin, Ahmad Razali Ishak, Jeyanthini Sathasivam

**Affiliations:** ^1^Department of Public Health Medicine, Faculty of Medicine, Universiti Teknologi MARA, Selangor, Malaysia; ^2^Training Management Division, Ministry of Health, Johor Bahru, Malaysia; ^3^Centre for Environmental Health and Safety, Faculty of Health Sciences, Universiti Teknologi MARA, Selangor, Malaysia; ^4^Public Health Division, Johor Health State Department, Ministry of Health, Johor Bahru, Malaysia

**Keywords:** indoor air quality, determinants, factors, building design, building operation, occupant factors, hospital, review

## Abstract

Indoor air quality (IAQ) has recently gained substantial traction as the airborne transmission of infectious respiratory disease becomes an increasing public health concern. Hospital indoor environments are complex ecosystems and strategies to improve hospital IAQ require greater appreciation of its potentially modifiable determinants, evidence of which are currently limited. This mini-review updates and integrates findings of previous literature to outline the current scientific evidence on the relationship between hospital IAQ and building design, building operation, and occupant-related factors. Emerging evidence has linked aspects of building design (dimensional, ventilation, and building envelope designs, construction and finishing materials, furnishing), building operation (ventilation operation and maintenance, hygiene maintenance, access control for hospital users), and occupants' characteristics (occupant activities, medical activities, adaptive behavior) to hospital IAQ. Despite the growing pool of IAQ literature, some important areas within hospitals (outpatient departments) and several key IAQ elements (dimensional aspects, room configurations, building materials, ventilation practices, adaptive behavior) remain understudied. Ventilation for hospitals continues to be challenging, as elevated levels of carbon monoxide, bioaerosols, and chemical compounds persist in indoor air despite having mechanical ventilation systems in place. To curb this public health issue, policy makers should champion implementing hospital IAQ surveillance system for all areas of the hospital building, applying interdisciplinary knowledge during the hospital design, construction and operation phase, and training of hospital staff with regards to operation, maintenance, and building control manipulation. Multipronged strategies targeting these important determinants are believed to be a viable strategy for the future control and improvement of hospital IAQ.

## Introduction

Air pollution significantly impacts human health and is regarded as the world's largest single environmental health risk by the World Health Organization (1). The indoor pollutant levels can be up to five times, or even 100 times, higher than outside pollutant levels, giving rise to significant concern as the average person typically spend ~90% of their time indoors (2). In recent years, the spread of the influenza A (H1N1) flu and severe acute respiratory syndrome (SARS-CoV) as well as the newly emerged severe acute respiratory syndrome coronavirus 2 (SARS-CoV-2) has been associated with airborne transmission (3, 4). This has brought IAQ into the limelight, notably in healthcare settings (5), and prompted a reconsideration of hospital IAQ and strategies for removing, diluting, and disinfecting harmful organisms from the hospital environment to prevent outbreaks (6). Reasonable air quality control in hospital facilities is not only vital for infection prevention considering patients with weakened immune systems (7), it is also central toward ensuring healthcare workers' optimal health, productivity and well-being (8).

Consequently, it is important to recognize the primary sources of hospital indoor pollutants to develop solutions for improving hospital IAQ. Indoor environments are complex ecosystems in which the interactions between humans, microorganisms, the physical environment, and architecture are intricate (9), and poor IAQ can be caused by a multitude of factors. On top of this, the elaborateness of hospital facilities is in itself well established due to their dimensions, advanced technologies for functioning, and 24/7 operability (10). The functions of different healthcare departments and rooms will determine the indoor air pollutants in their respective indoor air environment (7, 11). Many hospital spaces and processes have very specific requirements for ventilation (12). Moreover, differences in hospital occupants (patients, healthcare personnel, visitors) underlying health status and the variety of technologies applied in a hospital make the hospital microclimate more convoluted than other public utility buildings, as noted by previous studies (7, 13, 14).

Given the complexity of hospital IAQ and the urgent need to prevent the spread of infectious disease, a greater understanding of hospital IAQ and its determinants is needed. In this regard, the main factors influencing hospital IAQ, as synthesized and adapted from the findings/framework published by Gola et al. (12), Nasir et al. (15), and Korsavi et al. (16) can be classified into four broad categories including: (1) contextual factors on the macro level such as season and climatic conditions and micro level such as regional temperature, (2) building design factors such as room dimensions and finishing materials, (3) building operation factors such as management and maintenance activities for ventilation and hygiene, and (4) occupant factors such as occupant health status, behavior, and activities. Various elements of the hospital-built environment including building design, building operation, and occupant-related factors that can significantly impact the spread of pathogens (source, host, transmission pathway) are potentially modifiable, and thus amenable to prospective IAQ interventions. However, evidence in relation to these factors are limited as previous published reviews on IAQ have focused on other settings, including public utility buildings (7), residential and commercial buildings (17), and schools (18). From this perspective, this mini-review updates and integrates findings of previous literature to outline the current scientific evidence on the relationship between hospital IAQ and building design, building operation, and occupant-related factors, highlight the limitation of existing literature, present the relevant public health challenges, and propose recommendations to tackle this issue.

## Methods

We performed a literature search of publications written in English indexed from inception to October 2022 in PubMed and Scopus databases. Using various combinations of the keywords “hospital indoor air quality”, “building design”, “building operation” and “occupant factors”, any studies presenting findings on building design, building operation, and occupant-related factors of hospital IAQ were included. Searches through the references of articles retrieved were also performed to include relevant studies. A narrative review of 52 papers published between 1982 to 2022 was then performed, and the main findings were summarized in Table 1. Such reviews are believed to be helpful in presenting an up-to-date, broad perspective on the given topic (19).

**Table 1:** Summary of previous studies' main findings on factors related to hospital IAQ.

**Factor(s)**	**Main findings**
**A. Building design**	
Ceiling height	• Insufficient ceiling height leads to substandard total airflow rates
Door design	• Hinge door opening result in larger air volume exchange compared to sliding door
Ventilation system	
1. Mechanical vs. natural 2. Centralized vs. non-centralized 3. Outdoor air intake	• Mechanical ventilation lowers total bioaerosol level more effectively compared to natural ventilation • Centralized ventilation more effective control for chemical and particle contaminant compared to non-centralized ventilation • Ventilation system outdoor air intake influence the total bacterial load in indoor air
Construction and finishing materials	• Mold can grow in building materials • VOCs, mineral fibers, heavy metals, and radon can be released from building and finishing materials • Type of mirrors used for window/door influence indoor air temperature and moisture content and can promote mold growth
Furnishing	• Building furnishings can act as barriers that cause pollutant accumulation • Carpet may accumulate dust and microbes
**B. Building operation**	
Ventilation system maintenance	• Poorly maintained and inefficient ventilation system aid in infectious disease transmission
Hospital hygiene maintenance	• Irregular cleaning leads to PM build up • Cleaning and disinfectant activities may facilitate mold growth and release VOCs • Vacuum bags can accumulate microbes and mold
Access control for personnel, patients, and visitors	• Concentrations of bioaerosols, PM, and CO_2_is linearly related to number of room occupant • Overcrowding leads to poor IAQ
**C. Occupant-related factors**	
Occupant activities	• Occupant activities produce CO_2_, PM, chemical pollutants, and bioaerosols
Medical activities	• Certain medical therapies/activities may release bioaerosols, radon, NO_2_, anesthetic gases, phthalates, and BTEX compounds
Adaptive behavior	• Adequate operation of windows can erase accumulated CO_2_ concentration in the room • Appropriate manipulation of building envelope and air purifiers can lower indoor air pollutant levels

BTEX, benzene, toluene, ethylbenzene, and xylene; CO_2_, carbon dioxide; IAQ, indoor air quality; NO_2_, nitrogen dioxide; PM, particulate matter; VOC, volatile organic compounds.

### IAQ and building design

**Figure 1:**
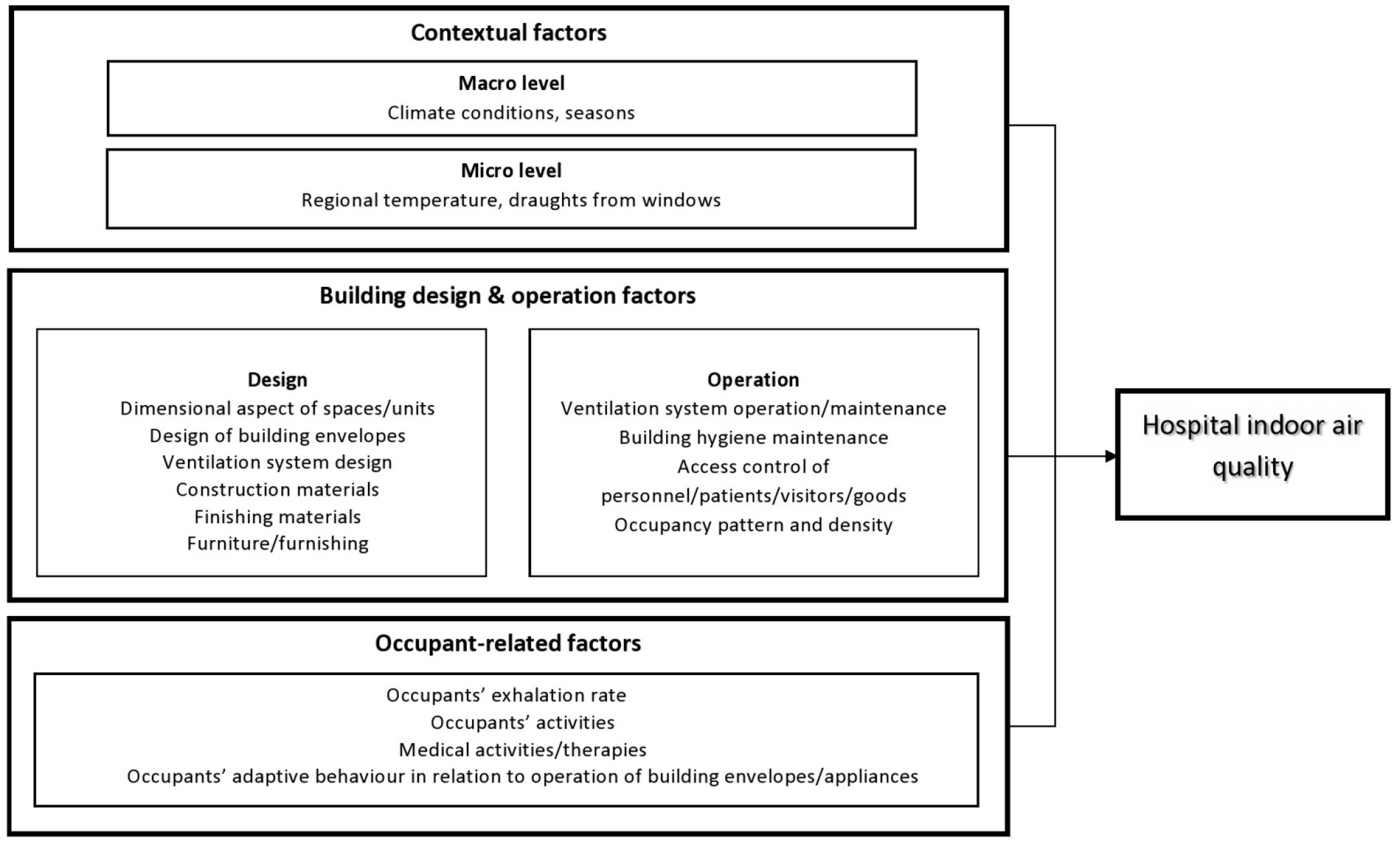
Determinants of hospital indoor air quality based on previous studies.

Building design refers to the description of a building represented by its detailed plan and specifications (20). Within the hospital setting, these include dimensional aspects of spaces, design of building envelopes (windows, doors), ventilation system design and outdoor air intake, construction and finishing materials, and furnishing (Figure 1).

Various hospital design aspects, including space dimensions, building envelope design, and ventilation system design and outdoor air intake were shown to influence hospital IAQ. Buildings with insufficient ceiling height were reported to have substandard total airflow rates (21). Isolation room IAQ were impacted by door design, as the greater flow generated from the opening of hinge door compared to sliding door resulted in a larger air volume exchange (22). In terms of ventilation systems, evidence suggest that hospitals fixed with sophisticated mechanical ventilation systems with higher air changes per hour, good directional flow, and filtration systems could keep its indoor air total bioaerosol concentrations at a lower level compared to hospitals with natural ventilation systems (23). Indeed, ventilation source and the resultant air flow rates, relative humidity, and temperature were observed to be significantly correlated with the diversity and composition of indoor bacterial communities, in which mechanically ventilated rooms were reported to contain less diverse microbial communities compared to window-ventilated rooms (14). Meanwhile, hospitals with centralized air conditioning and mechanical ventilation (ACMV) systems have been shown to have better indoor air control for chemical and particle contaminants compared to hospitals with non-centralized ventilation systems (24). Besides the design of ventilation system itself, its outdoor air intake can also influence hospital IAQ as previous studies had indicated that the total bacterial load detected in heating, ventilation, and air-conditioning (HVAC) system filters were higher in urban health facilities compared to rural ones, which was suggested to be due to the proximity to adjacent buildings and roadways (25).

The choice of construction materials, finishing materials, and furnishing has also been shown to influence hospital IAQ. A study in a hematology ward revealed that airborne Aspergillus fumigatus was caused by the insulating material in ceiling tiles (26). Indoor fungi have been observed to grow in building materials, especially in wetted wallpapered gypsum board and on the surface of ceiling tiles (27). Building materials, finishing materials, and furniture such as resilient floorings, paintings and coatings, sealants, varnishes, and adhesives contain volatile organic compounds (VOCs), fibrous insulations may release mineral fibers into the air, and heavy metals can be found in paint and varnishes and are used as stabilizers in vinyl plastic materials as well as radiation shielding (12, 28). Moreover, radon can be emitted from finishing materials and materials used during the construction phase (12). The type of mirrors used for window/door construction influence indoor air temperature and moisture content (28), which may lead to mold growth. In addition, building furnishings such as tables and equipment can act as barriers, causing pollutants to accumulate in the indoor air (24). Carpets may accumulate dust and microbes, and previous studies have demonstrated that a variety of bacteria and fungi can be aerosolized from these microbial reservoirs (29).

### IAQ and building operation

Building operation comprises a broad spectrum of services, capabilities, procedures, and technologies necessary to ensure that the built environment performs the functions for which it was designed and developed (30). Within the hospital settings, these include ventilation system operation and maintenance, building hygiene maintenance, access control for personnel, patients, visitors, and goods, and other maintenance and operational strategies (12) (Figure 1).

Ventilation system operation and maintenance plays a major role in hospital IAQ and a plethora of studies exists to demonstrate that it is critical toward controlling air-exchange rates, chemical and particle contaminants, excessive carbon dioxide levels, and occupant comfort levels (23, 24, 31, 32). Poorly maintained and inefficient ventilation systems, with contaminated ductwork and air supply, have been shown to aid in transmitting airborne infectious pathogens within hospital indoor environments (33–36). Of recent, evidence suggests that the transmission of Coronavirus Disease 2019 (COVID-19) infection is possible through heating, ventilation, and air-conditioning (HVAC) systems in built environments, and that the HVAC systems may aid its spread (34, 36). Indeed, according to the systematic review conducted by Aghalari et al. (33), the detectability of the SARS-CoV-2 in hospital wards and intensive care unit (ICU) indoor air samples were reported to be influenced by the efficiency and functionality of ventilation systems. In many cases, poor building ventilation performance has been the cause of inappropriate building ventilation and had resulted in poor IAQ within the hospital facility (31, 37).

Hospital hygiene maintenance also substantially influence IAQ by influencing the indoor pollutant levels (21, 31). Irregular cleaning lead to the continuous deposition of particulate matter (PM), and as its level increase, become re-suspended due to the movement of building occupants (38). In healthcare facilities, cleaning and disinfectant activities of equipment, furniture, floors and walls are vital even if dilution ventilation, source management, and design intervention have all been utilized optimally to control infectious aerosols (12). Paradoxically, the same activities may also increase humidity and facilitate the growth and survival of microorganisms, as well as increase the levels of total VOCs (12). Similarly to poorly maintained ventilation systems, vacuum bags in vacuum cleaners may also accumulate bacteria, mold, endotoxins, and allergens (39). As such, incorrect use of cleaning products when coupled with inadequate ventilation system result in poor hospital IAQ, as highlighted by previous studies (40).

Access control for personnel, patients, and visitors also influence hospital IAQ, as it impacts building occupancy density which has been found to attribute to the deterioration of a building's IAQ (41). Heo et al. (42) indicated that a building with a higher occupancy number possessed elevated levels of PM, bacterial, and fungal concentration. According to the study published by the authors, the increase in the number of people in a given chamber resulted in a significant increase in concentrations of bioaerosols, 1.2–3.5 times higher than those under vacant condition (42). Indeed, according to Verde et al. (43), quantitative evaluation of indoor and outdoor microbial load had indicated significantly lower levels outdoor compared to indoor, and that the maximum values for bacterial concentrations were found in rooms with higher occupancy. Meanwhile, previous studies have also demonstrated that particle and carbon dioxide concentrations were linearly related to the number of ICU occupants (44). In a study of a hospital outpatient department, overcrowding and inadequate ventilation were observed to contribute to poor IAQ in the said department (45).

### IAQ and occupant-related factors

Occupant-related factors encompasses occupants' activities, movement, adaptive behavior, and time spent in the room (16). Within the hospital setting, occupants' activities also include medical activities/therapies and the operation of medical equipment (12) (Figure 1).

Evidence suggests that hospital occupant activities produce indoor air contaminants such as PM, chemical pollutants, as well as bioaerosols (16). Occupants' breathing itself produces carbon dioxide, the generation of which is influenced by the total number of occupants, age, activity level, body surface area, room indoor temperature, and metabolic rate of each occupant (16). Actions such as talking, sneezing, coughing, walking, washing, and toilet flushing generate airborne biological contaminants in indoor environments (32, 42, 46). According to previous studies, the concentrations of bioaerosols generated by occupants showed significant variations according to the activities inside the room (standing, talking vs. moving), where the concentration was observed to be 1.2–4.5 times higher under moving activity compared to other conditions (42). In addition, occupants may also transport airborne pathogens through their clothes or resuspend PM and microbial materials from the floor by walking (47). In this regard, increased human movement were reported to speed up the spread of pollutants in a hospital waiting area (48), whereas the levels of PM were found to increase significantly during hospital visiting hours (49).

In relation to activities/therapies performed specific to hospitals, washing hands in sink, using medical sprays, performing nebulization therapy, bed making, and cleaning activities have been shown to influence particle levels in indoor air (50). Nebulization therapy in particular has been shown to result in large microbiological peaks during administration (51). Radon may be used during medical treatments for cancer and other diseases, such as radon baths to control autoimmune diseases (52), which may impact IAQ. Nitrogen dioxide can result from the use of anesthetics and medicines in hospital settings (53). Anesthetic gases used in operation theaters and disinfection gases used for cold sterilization of surgical utensils may exceed occupational exposure limits (OELs) if the devices and installations used for extracting such gases from the hospital environments are old, as may the case in poorer countries (7). Polymer materials and plastifiers including blood bags, plastic infusion bags, rubber tubing, and injectors may emit phthalates, which may be found in high concentrations in pharmacies and transfusion rooms (54). Besides that, the use of electronic devices such as copiers, printers, and computers that are used for hospital administration work may emit benzene, toluene, ethylbenzene, and xylene (BTEX) compounds (55), which are highly toxic to hospital users.

Occupants' adaptive behavior, defined as “observable actions or reactions of a person to adapt to ambient environmental conditions” (56), may also impact hospital IAQ. These include behavior such as the frequency and timing of operating building envelopes and appliances (windows, doors, air-conditioners, and fans). Evidence in relation to the impacts of occupants' adaptive behavior in hospital facilities are limited, but previous studies conducted in other settings have shown that “good practice” such as adequate operation of windows to erase accumulated carbon dioxide concentrations in the room leads to improved IAQ (16). According to a study conducted in a childcare center, occupants' behavior such as opening or closing windows/doors and utilizing air purifiers can affect the air exchange rate and raise or lower indoor air pollution levels (46). Occupants' behavior such as door opening may also impact IAQ, as previous studies have shown that exchange volume increased linearly with door opening speed, hold open time, and total cycle time (opening, hold open, closure) (57).

## Limitations of the existing literature

Most research on IAQ have concentrated on hospital areas with specialized conditions, such as the ICU, wards, operation theaters, and isolation rooms (11, 33, 35, 58). Meanwhile, common areas like the hospital outpatient departments are understudied and not evaluated well enough regarding their IAQ performances and the limiting and facilitating factors of having good IAQ (48, 59). In addition, the scientific literature presently does not provide many references in relation to important hospital IAQ determinants, such as building design aspects (dimensional aspects, room configurations, building materials, ventilation practices and technologies) and occupants' adaptive behavior (12, 17, 60). In relation to design factors, previous studies have focused on the minimum requirements for hospital spaces based on regulations, with no specific evidence outlined for space dimensions and configurations (12). Building materials contribute greatly to elevated VOC levels in buildings, yet few studies report on indoor materials when discussing IAQ (17). With regards to ventilation system, air filtration is a vital approach to controlling the spread of respiratory infectious disease, but evidence to support current practice is lacking and more research on air filtration and recirculation in healthcare facilities is warranted (6). Further exploration of ventilation strategies (mechanical, displacement, hybrid), room envelope conditions, and location of diffusers and exhaust ports is also needed to determine the airflow distribution and ventilation effectiveness for a given spatial domain (61). In this regard, mixed-mode ventilation that combines both natural and mechanical approach to optimize energy consumption and thermal comfort is ideal, but its application and control strategies require further investigation (62). Similarly, the insufficient evidence on the safety and efficacy of indoor air disinfection and purification technologies such as Ultraviolet Germicidal Irradiation limits its use and require further studies (37). Lastly, occupants' adaptive behavior were observed to impact IAQ more significantly compared to other determinants such as occupancy pattern and density (16), yet studies focusing on adaptive behavior within hospital settings are lacking (60). In particular, research on adaptive behavior should be expanded to consider integrated aspects of comfort (thermal, visual, acoustic, and air quality), as these aspects have been observed to significantly impact adaptive behavior (60).

In addition, defining and creating appropriate IAQ regulations and guidelines for various types of public utility environments remain a challenge, and more work needs to be done to ascertain what indoor environmental conditions should be met, and what parameters should be routinely monitored in healthcare facilities (7). While the OELs values in regulations and threshold limit values of the American Conference of Governmental Industrial Hygienists exist for industrial indoor environments, current legislations do not establish specifications for healthcare facilities specifically (63). It is possible to meet the quantitative criteria in accordance with legislations yet demonstrate the presence of toxigenic fungal species in hospitals, as evidenced by the study published by Viegas et al. (64). Presently in many countries, the current practice includes referring to analogous standards, such as the WHO guidelines for IAQ (63, 65). A recent systematic review on research trends on IAQ of healthcare units had indicated that non-compliance with this guideline was frequently reported (59), reinforcing the need for further research on barriers to guideline implementation and development of specific reference for healthcare facilities to improve this matter.

## Relevant public health challenges

Ventilation for hospitals has been argued to be challenging, as many spaces and processes have very specific requirements (12). In some cases, a mechanical ventilation system in a hospital may not always successfully provide optimal IAQ. For example, a hospital's indoor air CO level can still be present at a high concentration level even though the hospital is well equipped with ventilation system if the outdoor air CO level in the same area is already high (66). Moreover, even though mechanical ventilation system is effective in reducing the amount of bioaerosols in indoor air, Sham et al. (35) have identified that some fungal genera like Aspergillus, Cladosporium, and Penicillium were still frequently contaminating hospital indoor air. In addition, elevated concentrations of chemical compounds in indoor air remains a problem in many regions (7). The IAQ of a well-designed hospital may also deteriorate over time due to ongoing structure modifications to accommodate service expansions (67).

Moreover, while most developed countries adhere to IAQ regulations during the design and operation phase of building environments, many developing or underdeveloped countries do not (17). Indeed, although death rates from indoor air pollution have declined in almost all countries in the world, large differences in rates—>1,000-fold, persist between high-income and lower-income countries (68). According to the authors, there is a clear economic split in the issue of indoor air pollution, in which it is a problem that has been eliminated across high-income countries, yet remain a public health concern across lower-income countries (68). Previous studies suggest that in developing and underdeveloped countries, little attention has been given to identify and control the major sources of indoor air pollutants, and many buildings have been constructed based on other factors, including aesthetics, costs, and access to main street (28).

## Recommendations and future directions

In the wake of new and emerging infectious diseases, poor IAQ and airborne disease transmission can have severe implications for hospital users. A holistic approach to breaking the chain of transmission is fundamental toward controlling the spread of infectious disease in hospitals, and knowledge and practices from hospital environments, including building design, building operation, and hospital users' activities and adaptive behavior should be critically explored and applied. Indeed, a recent systematic review examining the trends in IAQ research has indicated that these elements are promising lines of research that may lead to IAQ improvement in healthcare facilities (59), and should be considered by prospective studies. Public health and policy action must be taken to ensure good IAQ in hospital environments to protect patients, visitors, and staff from occupational diseases and hospital acquired infections. First, hospital IAQ surveillance system should be implemented in which routine monitoring of IAQ and its pollutant levels are performed for all areas of the hospital building. This will enable the creation of a database that captures IAQ parameters and type and quantity of pollutants in various indoor environments, which may prompt appropriate legal regulations and guidelines aimed at improving hospital IAQ. Second, interdisciplinary knowledge needs to be considered during hospital design, construction, and operation, to support the needs of the users related to medical activities, issues related to nosocomial infections, design and application of operating systems and technologies to perform specialist healthcare disciplines, risk analysis of hospital units, and hospital management procedures to achieve acceptable residual risk (12). Third, hospital staff should be trained to use and manage spaces, including correct operation of ventilation systems and use of cleaning products, to minimize levels of indoor pollutants. To facilitate good practice of adaptive behavior, user friendly and efficient building controls which take into consideration the IAQ, comfort level and energy consumption of hospital users should be implemented. Multipronged strategies targeting these important determinants are believed to be a viable strategy for the future control and improvement of hospital IAQ.

## Author contributions

FI and ES conceptualized the topic, researched, wrote the manuscript, and including interpretations. ES, AI, and JS critically revised the manuscript for intellectual content. All authors read and approved the final version of the manuscript.

## Funding

This work was supported by the Geran Penyelidikan Dana UiTM Cawangan Selangor (DUCS) 4.0 [600-UiTMSEL (PI. 5/4) (015/2022)].

## Conflict of interest

The authors declare that the research was conducted in the absence of any commercial or financial relationships that could be construed as a potential conflict of interest.

## Publisher's note

All claims expressed in this article are solely those of the authors and do not necessarily represent those of their affiliated organizations, or those of the publisher, the editors and the reviewers. Any product that may be evaluated in this article, or claim that may be made by its manufacturer, is not guaranteed or endorsed by the publisher.
